# Examining the educational experiences of Behvarzes from the insufficient participation of some people in preventive measures against the COVID-19 pandemic: a lesson for the future

**DOI:** 10.1186/s12909-024-05752-0

**Published:** 2024-07-22

**Authors:** Zohreh Rajabi-Arani, Zahra Asadi-Piri, Fereshteh Zamani-Alavijeh, Fakhrosadat Mirhosseini, Shoaleh Bigdeli, Sucheta P. Dandekar, Fatemeh Bastami

**Affiliations:** 1 School of Behvarz Training Center, Kashan University of Medical sciences, Kashan, Iran; 2 Department of Health Management Policy and Economic, School of Public Health, Tehran University of Medical Science, Tehran, Iran; 3 Department of Health Education and Promotion, School of Health, Isfahan University of Medical Sciences, Isfahan, Iran; 4 Head of Anesthesia Department, Trauma Research Center & School of Allied Medical Sciences, Kashan University of Medical Sciences, Kashan, Iran; 5 Department of Medical Education, School of Medicine, Center for Educational Research in Medical Sciences (CERMS), Iran University of Medical Sciences (IUMS), Tehran, Iran; 6 Era’s Lucknow Medical College, Lucknow, India; 7 Social Determinants of Health Research Center, Lorestan University of Medical Sciences, Khorramabad, Iran

**Keywords:** Health care workers, Qualitative research, Iran, Preventive measures of COVID-19

## Abstract

**Background:**

This study aims to explore the experiences of Behvarzes regarding the reasons behind the insufficient participation of some individuals with the preventive protocols established during the COVID-19 pandemic.

**Methods:**

A qualitative study was conducted from July 2021 to December 2022 using the conventional content analysis method. Purposive sampling was employed to select 14 Behvarzes working in villages covered by Kashan University of Medical Sciences. Data were collected through semi-structured in-depth individual interviews and analyzed using conventional content analysis.

**Results:**

The study identified seven subcategories which were grouped into two main categories of reasons for inadequate compliance with health protocols by some individuals. These include: (1) Intentional non-compliance with preventive protocols, with the following subcategories: perceived obligation and adherence to social customs, denial of risk, belief in external health locus of control, and fear and distrust of prevention and treatment methods. (2) Unintentional non-compliance with preventive protocols, with the following subcategories: insufficient or contradictory information, negligence, and inevitability.

**Conclusion:**

The findings suggest that ensuring compliance with health guidelines is not a one-size-fits-all approach. providing empowerment and obstacle removal solutions to those forced to violate preventive protocols for various reasons are all critical components of successful interventions. Also, cultural familiarity can aid in the design of appropriate interventions to address these challenges.

## Background

The emergence of COVID-19, a severe respiratory infection with a viral source, in Wuhan, China in December 2019 marked the onset of a global pandemic with significant impacts on public health.The World Health Organization declared COVID-19 a public health emergency of international concern on January 30, 2020, and a pandemic on March 11, 2020 [1, 2]. The available literature indicates that a considerable proportion, specifically 80%, of COVID-19 cases are classified as mild, and even patients with moderate disease can be adequately managed through primary healthcare services [3]. This underscores the critical role played by health care workers (HWs) in effectively managing the pandemic. All local health system units, including personnel who did not have expertise in infectious diseases, joined the fight against COVID-19 to control the disease and care for infected patients [4–6].

Many of the HWs attempted to overcome the challenges regarding to COVID-19 with patience and professional dedication [7]. However, they encountered a notable proportion of individuals within the community who demonstrated inadequate awareness and limited participation in preventing the progression of the pandemic. These individuals exhibited avoidance towards learning and adhering to COVID-19 preventive protocols [8, 9]. Even if exhibited by a minority of individuals within the community, such behaviors have the potential to significantly contribute to the spread of the disease. As a result, HWs must be prepared to effectively address these challenges and promote maximum community participation in preventing further transmission [10]. Attaining a comprehensive understanding of the processes involved in HWs’ preparedness, their first-hand experiences, and the control measures can significantly enhance service providers’ ability to respond efficiently to sudden pandemics. This not only helps to increase public confidence in the health system [11], but also facilitates effective management of psychological pressures and associated problems experienced by HWs [12].

The World Health Organization has recognized Health Workers (HWs), known as Behvarz in Iran, as “native people of the village and trained local workers who provide primary health care to the rural community of Iran.” An Iranian Behvarz is a full-time employee of Iran’s health system, selected from their own community and place of residence, and stationed in rural health houses, which are among the most important health facilities in rural areas of Iran. Behvarzes in villages serve as managers of these rural health houses and are responsible for the health of the people in their region.

Despite severe shortages, Behvarzes have effectively controlled and prevented the Covid-19 crisis with the help of the community and guidance from health centers. They have been responsible for various tasks including environmental health services, visits to public places, food health preparation and distribution, control of gatherings and holidays throughout the year, participation in mourning ceremonies, and caring for Covid-19 patients. These responsibilities are distinct from those typically assigned to healthcare workers. [13]. As natives of the villages where they work, Behvarzes have a close relationship with the local population. They provide standard and effective services while being intimately familiar with the culture, behaviors, and beliefs of the community. This enables them to closely cooperate with the community to promote health and prevent diseases [14]. The cooperation with development programs from other sectors has contributed to the overall economic development of rural populations and has led to sustainable improvements in health indicators. The active provision of healthcare services by trained nurses plays a pivotal role in improving the health status of rural communities. Iran’s health and treatment network system, with nurses at its center, has achieved remarkable achievements [15]. Screening over 78 million people by both health workers and healthcare workers, and referring suspected cases to selected comprehensive health service centers and hospitals for Covid-19, stands out as one of the remarkable achievements of this program [16].

Considering that the problems and experiences faced by Behvarzes, as community health workers in rural areas of Iran, may be significantly influenced by their unique context, an in-depth inquiry is essential. Therefore, the main question of this research is: What experiences have Behvarzes gained in relation to prevention and control measures during the COVID-19 pandemic? What are the underlying structures and perceptions that have shaped these experiences? Given the dearth of information available in this area of research, a qualitative methodology was employed to conduct an in-depth exploration of Behvarzes’ experiences. Scholars suggest that under circumstances where the phenomenon being studied is not well understood, utilizing qualitative research methods may prove more efficacious in uncovering factors and obtaining comprehensive qualitative insights [17, 18]. Consequently, this study was conducted using a qualitative methodology with the objective of elucidating Behvarzes’ experiences related to the underlying rationales for inadequate participation in and adherence to the preventive protocols amidst the COVID-19 pandemic.

## Methods

### Research design

This study was conducted using the conventional content analysis method between 2020 and 2022, following approval and acquisition of the scientific code (400,034) from Kashan University of Medical Sciences in Kashan, a central county in Iran. The application of this approach is instrumental in gaining insight into phenomena for which theories are presently few and far between [19]. The current article focuses on a subset of the findings from a much broader study aimed at investigating the challenges faced by Behvarzes regarding the adherence of individuals to the established protocols and the remedies employed by Behvarzes in response. Due to the substantial volume of findings generated by the study, this article has opted to present only the challenges and their underlying reasons.

### Setting and participants

Participants were recruited from local health system units in Kashan, Iran, between 1 July 2021 and 15 December 2022. Behvarzes from the cities of Kashan, Aran, and Bidgol, which are covered by Kashan University of Medical Sciences, were selected. Preference was given to Behvarzes in disadvantaged or more populated areas, as well as those whose activity reports were accessible. Interviews were conducted based on the research objectives, utilizing a semi-structured questionnaire prepared specifically for this purpose. Participants were encouraged to freely express their thoughts and experiences without any word limit. Some Behvarzes shared poignant memories during the interviews. The duration of each interview ranged from one to two and a half hours, with an average of two hours. Interviews took place either at the health house in the village where Behvarzes were stationed or at the Behvarzes training center, chosen based on participants’ preferences and convenience.

The eligibility criteria for study participants included active employment as a Behvarz in rural areas overseen by Kashan University of Medical Sciences, a minimum of one month of experience in the prevention and control program of the COVID-19 pandemic, and an interest in sharing their experiences and participating in the study. Purposive sampling was employed to recruit participants with maximum variation in terms of their education level, geographical location, and work experience.

The interview guide comprised the following questions:

1. What experiences and challenges have you encountered in caring for patients with COVID-19?
2. What experiences and challenges have you faced regarding household screening and active detection of COVID-19?
3. What experiences and challenges have you encountered in providing environmental and professional health services for the prevention and control of COVID-19?
4. What measures and challenges have you faced in controlling gatherings and various festivals and ceremonies?
5. Have you encountered experiences and challenges in providing education and information services to people for the prevention and control of COVID-19?
6. What challenges have patients experienced in controlling and preventing COVID-19?

### Data collection

Data collection was accomplished using the semi-structured in-depth interview technique, with consideration given to triangulation [20]. Participants were selected based on various factors aimed at identifying Behvarzes with diverse experiences and unique perspectives. These factors included job prospects, the size of the rural population they served, their proximity to urban areas, previous patterns of cooperation or lack thereof within their respective villages, and the prevalence of endemic diseases compared to other nearby villages.

To ensure the study was conducted professionally and respectfully, advance coordination efforts were made to establish a comfortable environment for all participants. The timing and location of the interviews were determined by the Behvarzes themselves, and the duration varied depending on circumstances. The interviews began with an expression of gratitude towards the Behvarz, followed by a request for explicit consent to commence recording, with reassurance that it would be promptly terminated at their discretion. This was followed by meticulous collection of demographic data on relevant parameters such as age, education, and marital status.

Following this, the main data collection phase commenced with open-ended inquiries within the domain of participants’ experiences, such as the prompt, “Please define the nature of care you provided to different groups of COVID-19 patients.” This methodology is consistent with established psychological research practices aimed at achieving a holistic understanding of participants’ perspectives [21]. Probing questions, such as “What obstacles did you encounter in managing and mitigating the spread of COVID-19?”, along with requests for further elaboration on ambiguous remarks relevant to the research objectives, were employed to gather richer data. These interview techniques were utilized to ensure comprehensive exploration of the subject matter. If respondents mentioned insufficient participation of target groups in adopting health behaviors for the prevention and control of the Covid-19 epidemic, they were asked to provide further explanations and identify related issues. Explanations, factors, and reasons mentioned were extracted, leading to the formation of primary codes, and subsequently, secondary codes. These codes were then categorized under a larger category titled “Behvarzan’s experience of insufficient participation of some people in preventive measures against the Covid-19 epidemic” in the article.

After each interview, the contents were transcribed and analyzed. If any ambiguous information was identified post-interview, participants were contacted for clarification. A total of 14 Behvarzes were interviewed, with each interview lasting between 60 and 120 min. Data collection continued until data saturation was reached, ensuring comprehensive coverage of the topic.

### Data analysis

Following the inductive content analysis process [22],each interview was meticulously transcribed, and a thorough line-by-line review was conducted to identify meaning units and extract primary codes from them. Subsequently, primary codes were named and categorized using constant comparison, with similarities and differences serving as criteria. If necessary, interview questions were modified in alignment with the established codes and categories, while remaining mindful of the research objectives. Moreover, as new data emerged from subsequent interviews, existing categories were either completed or subdivided to form new categories. This iterative data analysis process continued until data saturation [12] was achieved, along with the identification of key themes. To aid in the organization of codes, subcategories, and categories, MAXQDA software (VERBI GmbH, Berlin, Germany; version 10) was utilized.

### Trustworthiness

To enhance the scholarly robustness of the findings, the standards and techniques proposed by Lincoln and Guba, as cited in Polit and Beck [23], were employed. To bolster the transferability of findings, demographic variables such as gender, age, education level, and work experience were presented via tables to provide a contextualized understanding of the primary codes, categories, and concepts obtained. This facilitated readers’ evaluation of the results’ applicability within comparable settings or groups. To ensure the credibility of the data, sustained engagement was employed. Additionally, three members of the research team independently undertook the textual coding process and identified new codes. These codes were later consolidated through collaborative efforts to finalize them.

The researchers conducted interviews with participants selected to represent maximum variation in terms of geographic regions within the county, work experience ranging from less than 15 years to 30 years, and education levels from high school diploma to university bachelor’s degree. These interviews were facilitated by two proficient research assistants with significant experience in Community Health Worker (CHW) activities. The research assistants approached the interviews with open-mindedness and employed bracketing techniques to suspend their personal opinions. Interviews were conducted at either health houses or Behvarz education centers, based on the preferences of the participants. Health houses are primary healthcare facilities staffed by Behvarzes, providing fundamental medical services and health education to individuals in rural and underserved areas of Iran.

Furthermore, the data coding process, interpretation, and categorization were reviewed by three experienced qualitative research specialists, who provided their expert opinions. This thorough review process ensured the accuracy and validity of the study’s findings.

## Results

All participating Behvarzes were employed at health houses within the rural areas of Kashan County. The gender distribution among participants was 57% male and 43% female. In terms of educational attainment, 29% held a bachelor’s degree, 7% held an associate degree, 50% possessed a high school diploma, and 14% had dropped out of school. The maximum experience of participating Behvarzes ranged between 26 and 30 years. Table 1 presents the demographic profile of the study participants.

**Table 1:** Demographic profile of participating Behvarzes in interviews

Title		Count [14]	Percentage
Gender	Male	8	57
	Female	6	43
Education Level	School Dropout	2	14
	Diploma	7	50
	Associate	1	7
	Bachelor’s	4	29
Work Experience (year)	Under 15	3	21
	15–25	5	36
	26–30	6	43
Location of Interviews	Health House	13	92.86
	Behvarz Education Center	1	7.14

The analysis of Behvarzes’ experiences revealed a substantial number of 535 primary codes, 24 subcategories, and 7 secondary categories. These findings were ultimately organized into two main categories addressing the underlying reasons for individuals’ inadequate adherence to preventive protocols: (1) intentional non-compliance, and (2) unintentional non-compliance (see Table 2).

**Table 2:** Underlying reasons for individuals’ inadequate adherence to preventive protocols

Main Categories	Secondary Categories	Subcategories
1) Intentional non-compliance	Perceived obligation to adherence to social customs	Resistance to changing the approach to handling and caring for patients
		Insistence on using common customs to show respect
		Resistance to changing job relationships
		Insistence on participating in cultural ceremonies
		Adherence to false traditional medicine
	Denial of risk	Low perceived likelihood of contracting the disease
		Low perceived severity of disease
	Belief in external health locus of control	Fate
		Luck
		Others
		God
	Fear and distrust of prevention and treatment methods	Fear and distrust of medical procedures
		Fear of vaccine risks
		Fear of social stigma
2) Unintentional non-compliance	Insufficient or contradictory information	Insufficient information and skills
		Confusion and contradiction
	Negligence	Remissness
		Forgetfulness
	Inevitability	Urgency and unforeseen matters
		Compulsion (due to circumstances)
		Lack of choice
		Insufficient remote access to essential goods and services
		Insufficient access to health goods and services
		Insufficient support for quarantine

### Intentional non-compliance with preventive protocols

Based on the insights shared by Behvarzes, it was inferred that a proportion of individuals during both the initial stages and ongoing phases of the pandemic consciously chose not to comply with established health protocols. Intentional non-compliance emerged as a prominent concept derived from four distinct secondary categories: perceived obligation and adherence to social customs, denial of risk, belief in external health locus of control, and fear and distrust of prevention and treatment methods.

#### Perceived obligation and adherence to social customs

Two aspects of this challenge included **“resistance to changing the approach to handling and caring for patients”** and **“insistence on using common customs to show respect.”** Behvarzes involved in the study noted that certain ill and suspicious individuals failed to comply with prescribed separation and quarantine measures due to their cultural beliefs. They even interacted with other individuals, such as family members and acquaintances, without masks. One participant mentioned, *“In our village*,* it’s customary for the patient’s relatives and neighbors to visit and offer help and not leave them alone. Even though we trained them*,* some didn’t follow the separation and quarantine measures at all. Because they considered wearing a mask near the patient an insult and believed it to be unethical*” (P12). The concept of **“resistance to changing job relationships”** was also evident from the experiences shared by a few Behvarzes. One factor contributing to resistance in workplace compliance with distancing and masking protocols is a strong adherence especially shopkeepers, traditional practices.

According to the accounts of Behvarzes, **“insistence on participating in cultural ceremonies”** was another contributing factor in individuals’ reluctance to comply with preventive health measures for COVID-19. They reported that people’s involvement in communal gatherings, including weddings, funerals, and religious ceremonies, remained a significant challenge despite repeated warnings and advice. This phenomenon, as observed by the study participants, was attributed to the high degree of interrelatedness and familiarity among families and the significant value placed on familial ties, serving as a contributing factor to the increase of disease transmission. *“One of our challenges was funeral processions. Whenever someone died*,* people would gather for the burial ceremony. We closed the large cemetery gate to prevent gatherings*,* but during one ceremony*,* a large crowd broke the door to enter the cemetery”* (P8).

Another participant expressed, *“From the beginning of the crisis*,* we closed all public places like schools*,* mosques*,* confraternities*,* sports fields*,* public baths*,* Quranic sessions*,* and religious ceremonies*,* but canceling funeral processions was one of the toughest and most bitter things we had to do because it was really difficult to persuade mourning families not to hold the procession. Because everyone in our village is related and familiar with each other and a large population would attend”* (P13).

**“Adherence to false traditional medicine”** was identified as another contributing factor.Participants stated that belief in traditional medicine among the public has led to the proliferation of unscientific methods in disease prevention and different recommendations by adherents of this medicine to avoid vaccination. One participant stated, “*False traditional medicine was a significant issue. People in our village strongly believe in traditional medicine. Individuals without any expertise would introduce medications solely for their own financial gain. They would advise patients against using certain medications*,* claiming them to be harmful. They’d discourage following the guidance of Behvarzes or doctors*,* even asserting that wearing masks could worsen the situation. They advocated for holding traditional ceremonies instead of adhering to medical advice. Unfortunately*,* some people followed such advice.”* (P11). Another Behvarz explained, *“Our village council believed in traditional medicine*,* and he would say wear a necklace of Espand and garlic and you will be safe from Corona. He kept going to the Hussainiya and mosque to preach. No matter how much we explained to him*,* it was useless. Until his mother got sick and traditional medicine failed to treat her. When he saw his mother was wasting away*,* he was finally forced to take her to the hospital on the doctor’s orders. Then he realized that Corona really exists.”* (P8).

#### Denial of risk

Participants reported that despite providing training and warnings, some individuals perceived their risk of contracting the disease to be low. This led to the identification of the concept of **“low perceived likelihood of contracting the disease”.** Additionally, Behvarzes believed that some individuals did not acknowledge the severity, risks, and potential complications of the disease, known and unknown, instead viewing it as a common cold. This observation also led to the identification of the concept of **“low perceived severity of disease”**. Consequently, the combination of low perceived probability of contracting the disease and its potential severity led to neglect among some individuals. Also, negligence and indifference resulted in the reopening of clubs, Hussainiyas (religious centers), and mosques. One participant explained, “*One of the problems we faced in the village was the lack of response from some individuals to our phone calls regarding the first to third screening stages. Sometimes*,* we even visited their houses in person*,* but they wouldn’t answer us properly or would say*,* ‘Why do you keep calling? We’re sure we won’t catch this disease.’ Some even said*,* ‘Why are you taking this disease so seriously? It’s just like any other disease.’ Even when they had symptoms*,* they wouldn’t inform us.”* (P10). Another Behvarz added, *“Since the middle of last year*,* after the waves of Corona*,* it became normalized for people. They continued with their travels and disregarded our instructions. If they contracted Corona*,* they wouldn’t adhere to social distancing measures*,* reasoning that ‘Many people have had Corona and nothing serious happened to them. They only experienced mild cold symptoms for a few days.’ Or they themselves had contracted the virus and experienced a very mild illness*,* leading them to underestimate the risk. They became lax in their precautions; it all became routine for them. They roamed the village without masks and attended all ceremonies.”* (P9).

#### Belief in external health locus of control

Drawing from the experiences of Behvarzes, it was found that certain individuals attributed the underlying cause of their illnesses to external factors such as **“fate**,**” “luck**,**”** the will of **“God**,**”** or even “others,” without assuming responsibility for their own health. This perspective not only undermined the importance of health instructions but also posed a significant obstacle to disease control efforts across the entire village. One Behvarz explained, *“There is a percentage of people who resist. They believe they have no control over whether they remain healthy. They argue that our health and sickness are not within our control but are determined by fate and luck. Some also believe we are helpless; only God decides whether we remain alive*,* healthy*,* or fall ill and die.”* (P9).

#### Fear and distrust of prevention and treatment methods

The examination of Behvarzes’ experiences revealed that **“fear and distrust of medical procedures”** may have influenced certain individuals to refrain from seeking care and treatment, despite experiencing symptoms. This behavior resulted in a deterioration of their condition and facilitated the transmission of the disease to others in their proximity. Moreover, Behvarzes acknowledged that the presence of fear and mistrust among some individuals even led to non-cooperation. For instance, a Behvarz provided an account of their experience as follows, *“At first*,* a few people would say that if you go to the hospital*,* they will kill you. Even this old lady whom I told to get hospitalized; I had gone to her house several times to give her a serum*,* but she refused to go to the hospital. In the end*,* the poor woman passed away at her house one night. Nobody would come to help; I was all alone*,* there was no one. And I was there until 12 a.m. when her grandchildren called for a car. They said we’re afraid to come closer. Eventually*,* we put the deceased in the back of a Nissan car (Behvarz’s sorrow about the situation). I sent them to Dar al-Salam at midnight.”* (P8). Another manifestation of fear experienced by individuals concerned the **“fear of vaccine risks”**. “Mistrust of preventive methods along with fear of vaccine risks and complications led to people’s resistance towards prevention and initiation of the vaccination process … At the beginning of the vaccination program, some people were supporters of traditional medicine. They said, ‘You want to kill the elderly aged 80 years old and above. Reduce their population. Why won’t you start the vaccination with people under 80 years old?’ Such talk spread fear, stress, and anxiety among people, which made some individuals less inclined to get vaccinated.” (P14). Similarly, another Behvarz expanded on the role of certain individuals in instigating mistrust and fear towards vaccination among the populace at the outset of the program.

Through qualitative analysis of Behvarz’s statements, it became evident that the **“fear of social stigma”**, the stigmatization of the disease itself, as well as negative and pessimistic perceptions, were pivotal factors in fostering individuals’ fear of ostracism, consequently dissuading them from disclosing their illness or that of their kin. This, in turn, facilitated the spread of the disease within the community. One participant explained, “*One afternoon*,* someone came and said*,* ‘The lady in that house is sick.’ I called the doctor*,* and together*,* we went to their house. We found she’d died. Her chest X-ray was suspicious of Corona and her lungs were completely infected. Her family had taken her to get a scan before but hadn’t taken it to the doctor. I closed the door to her room*,* and her sons and daughters came to me with horror and said*,* ‘Why are you doing this?’ I said*,* ‘Well*,* she’s suspicious*,* you shouldn’t go in.’ They started shouting*,* ‘Why would you say such a thing? Why do you accuse my mom? She didn’t have Corona!’ Because they stigmatized it and were afraid their reputation would be ruined. They still think that way.” The participant continued*,* “… Since the mourning ceremony was at their house*,* I asked the village head to arrange the disinfection of her house at night. I also called the council for social distancing during tomorrow’s lunch at the mourning ceremony. They all agreed*,* but none of them showed up. So*,* I gave one of the village boys gloves*,* masks*,* and a gown from the care kit. We disinfected everything. Then I saw that the village head and council member had also arrived*,* but they were standing far away. They were petrified.”* (P12).

### Unintentional non-compliance with preventive protocols

Another main category that emerged from Behvarzes’ experiences pertained to the phenomenon of unintentional non-compliance with health protocols among certain individuals. This category was derived from three distinct secondary categories, including “insufficient or contradictory information,” “negligence,” and “inevitability.”

#### Insufficient or contradictory information

The experiences of Behvarzes have also revealed that, in some cases, **“insufficient information and skills”** among certain individuals were barriers to controlling COVID-19. Participants believed that limitations caused by factors such as old age and low literacy levels led to individuals struggling to receive relevant information through educational media and acquire the necessary skills in face-to-face classes. The impact of these barriers was exacerbated by challenges among these groups in accessing virtual platforms and e-learning resources. Moreover, it was highlighted that the acceptance and awareness of individuals aged 80 years and above were inadequate, further complicating the understanding of educational materials and acceptance of the education. One Behvarz noted, *“Our issues were regarding the elderly because they were less careful; they didn’t know and couldn’t. But their illness was harder and more deadly. The number of elderly in our area is more than the young and the middle-aged populace. They were illiterate and did not have access to the virtual platforms*,* and yet we wanted to give them information. Some of them could not see and some were deaf or hard of hearing; this made our job much harder… Educating the non-native and immigrant individuals living here was also difficult. Everything was perplexing to them. I had to explain each topic loudly and several times. I was wearing a mask too*,* it was difficult. But I understood they were not receiving enough information.”* (P9).

The Behvarzes acknowledged that even some individuals who were both educated and in possession of smartphones failed to comply with health protocols. This was likely due to exposure to conflicting information. Behvarzes inferred that this group of individuals may have encountered a plethora of inaccurate or unhelpful information, which in turn led to confusion in decision-making and contributed to behavior contrary to health protocols. From this critical section of the participants’ experiences emerged the concept of **“confusion and contradiction”** as a reason for non-compliance during the COVID-19 pandemic.

#### Negligence

Based on the observations of the study participants, it appears that certain individuals were inclined to accept health protocols but simultaneously displayed a tendency towards neglect and procrastination in their actual adherence without any justifiable rationale. This group of experiences under scrutiny has been labeled as **“remissness”.** “… For example,” one Behvarze explained, *“a few people in our village were completely in favor of preventive protocols to control COVID-19 and even educated others and encouraged everyone to get vaccinated. But they didn’t make time for their own vaccination. They would say*,* ‘I can’t this week. I’ll go next week.’ They did not take it seriously. One day*,* I just took the cold box with me to their workplace or home and vaccinated them. These people are just this way. Their next appointment was the same; even though they had no pain or problem from the previous dose*,* they wouldn’t show up. It was like it wasn’t a priority.”* (P13).

Additionally, Behvarzes’ experiences revealed “**forgetfulness”** as another factor contributing to negligence among individuals who were in favor of vaccination. One participant said, *“… There were a few who forgot their vaccination appointment. They got their children vaccinated on time but were negligent about themselves. How could someone forget such an important matter? But some people were counting the days and eager to complete their vaccination as soon as possible.” The participant added*,* “I would ask them*,* ‘Why didn’t you wear a mask?’ and they’d say*,* ‘I forgot’!”* (P12).

#### Inevitability

Drawing from the accounts of participating Behvarzes, another concept named **“compelled exposure to crowded settings”** was identified, which revealed that some individuals were compelled to disregard health protocols in order to carry out essential tasks. For instance, one Behvarz explained their experience as follows, *“… In the early days of the pandemic*,* everyone was caught off guard. Offices were crowded and no plans had yet been made to put distance between visitors. People said that sometimes even during lockdown*,* to buy groceries or carry out other essential tasks they had to visit crowded places like doctors’ offices or judicial service offices and get in line with crowds*,* deliver their documents. Hence*,* certain circumstances led to people not complying with health protocols.”* (P12).

Additionally, some accounts indicated that **“insufficient remote access to essential goods and services”** and **“insufficient access to health goods and services”** were reasons for individuals’ non-compliance.

The next extracted category in this dimension was named **“insufficient support for quarantine”**. One participant explained, *“Some people didn’t have anyone. They would say*,* ‘If we stay quarantined*,* we and our families will starve to death. There is no one to help us.’” The Behvarz continued*,* “They were forced to act themselves to meet their needs. Due to the daily wages of most people in the village*,* they did not have a fixed salary. This was an important factor in the lack of compliance with quarantine in some healthy and even sick individuals*,* which was a challenge and obstacle for us Behvarzes in controlling the disease. For example*,* a taxi driver himself had symptoms and infected others in his car.”* (P3).

## Discussion

The COVID-19 crisis posed significant challenges for the healthcare system, prompting a reassessment of management strategies in Iran. Behvarzes played a crucial frontline role, drawing on their extensive experience to combat the pandemic. This qualitative study explores Behvarzes’ experiences with inadequate public participation in preventative protocols during the crisis. The findings categorize reasons for non-participation into intentional and unintentional non-compliance, which are obtained from 7 subcategories. In the following, the discussion about these two categories of obtained results will be discussed.

### Intentional non-compliance

The present study’s findings indicate that inadequate adherence to health education and protocols has posed several challenges in controlling COVID-19. This, in turn, has contributed to the spread of the disease and heightened mortality rates. Cultural attitudes and beliefs have played a role in people’s hesitancy to comply with disease prevention guidelines. Specifically, reluctance to isolate ill individuals, opting instead to visit them, insistence on attending cultural events (especially weddings and funerals) and religious ceremonies, and reliance on traditional medicine and home remedies seem to be deeply ingrained behaviors tied to culture that many individuals engaged in during the COVID crisis. According to Bijani et al.’s (2021) qualitative research based on senior managers’ experiences, cultural issues caused individuals to disregard protocols and attend family gatherings amid the COVID-19 pandemic. Additionally, people’s beliefs and strong desires to participate in congregational prayer and mourning for religious elders led them to take part in events even at the risk of illness or death [24, 25]. Other studies conducted in Iran have highlighted how disregarding government warnings concerning quarantine implementation and avoiding cultural and religious gatherings and travel posed significant challenges in managing the COVID crisis [25, 26]. Based on the findings of the present study, some people intentionally did not adhere to the adoption of preventive behaviors against Covid-19 due to four main reasons: **Adherence to social customs; Denial of risk; Distrust of prevention and treatment methods; Fear of social stigma**, which are listed below and have been discussed.

#### Adherence to social customs/dos and don’ts

Despite laws being enacted to prohibit participation in family gatherings, weddings, mourning ceremonies, cultural events, sports events, and other large-scale gatherings during the COVID-19 pandemic, the current study highlights that such interventions continue to face significant challenges. Culture has emerged as a critical factor shaping the course of the COVID-19 pandemic across various communities [27–29], and different countries have adopted diverse strategies to enhance public participation while simultaneously reducing cultural barriers. For instance, in the Indian state of Kerala, local voluntary committees and campaigns have been utilized to mobilize people for education and behavior monitoring related to gatherings, comings and goings, and quarantine. These committees have served as an additional set of eyes for health workers to monitor community behavior [30]. Moreover, research into the resilience of the healthcare system across 28 countries facing the COVID-19 crisis found that engaging and training local volunteers during epidemics fosters community participation and appropriate response to these crises [31]. Consequently, an understanding of unique cultural practices is crucial in designing and implementing effective interventions.

#### Denial of risk/low public trust

Based on the perspectives of study participants, insufficient adherence to preventive methods and health guidelines can be attributed in part to people’s denial of risk. In this research, it was observed that some individuals did not believe in the existence and complications of the disease and considered it to be a mere cold. Similar findings were reported in Nigeria where a study found that common misconceptions about COVID-19 presented a significant challenge for all participants, with some individuals believing that the disease only existed in other countries [32]. In Iran, the results of a qualitative study conducted in 2021 also indicated that limited belief and understanding of disease risk among both the public and officials impeded efforts to control the disease [19]. Likewise, Labbaf et al. (2020) observed a lack of sensitivity among managers towards COVID-19, which hampered crisis management at Tehran University of Medical Sciences [33]. These findings collectively suggest that some individuals may fail to acknowledge the reality of the disease until they themselves become infected, likely due to insufficient trust in official guidance and warnings.

Notably as shown by the evidence, many officials themselves did not take the crisis seriously in the early stages of the pandemic, which contributed to the neglect of necessary measures and ultimately reinforced public lack of trust in the existence of the disease. For instance, the absence of quarantine in Qom province, the first province in Iran to encounter COVID-19, reflects authorities’ initial lack of urgency. Raufi et al. (2020) similarly reported that delayed decisive action posed the greatest challenge in policy-making to combat COVID-19 [34], while countries successful in controlling the disease demonstrated quick action, prediction, and sensitivity to the crisis [35]. These findings suggest that a greater level of seriousness from authorities and policymakers could have resulted in a more serious attitude towards the disease among the general public. As such, it is critical for authorities and policymakers to prioritize crisis management and communicate up-to-date and credible information to build and maintain public trust. Appropriate education to build strong beliefs and structures for implementing and controlling communicated guidelines in pandemics could help involve people in controlling disease outbreaks.

#### Distrust of prevention and treatment methods

The lack of trust in disease prevention and treatment methods has been identified as a contributing factor to non-compliance with health protocols. This is consistent with the results of a different qualitative study, which highlighted that establishing trust and confidence among individuals in relation to government actions, such as quarantine measures, is a crucial element of COVID-19 crisis management. Additionally, the successful management of unwarranted fear or rumors at the community level has been shown to contribute to successful control of COVID-19, according to the same study [27].

#### Fear of social stigma

In the present study, it was observed that fear of social stigma has hindered individuals from seeking diagnosis and treatment. This finding is consistent with previous research by Ray et al. (2021), who demonstrated that fear of social stigma and resistance to interventions can impede disease control efforts [36]. Similarly, a study conducted in Nigeria revealed that social stigma towards COVID-19 patients resulted in many individuals concealing their illness when visiting healthcare centers, thereby making it challenging to identify cases in the community [32]. Consequently, the use of various interventions aimed at increasing people’s confidence in health recommendations, raising awareness, and changing attitudes towards the disease becomes particularly important.

Several studies have investigated different approaches to improving public trust and adherence to health guidelines. For example, Molineux et al. (2012) found that direct participation by customers, users, and the general public in providing healthcare can strengthen public trust and responsibility in health systems [37]. In another study, community and stakeholder involvement was shown to be crucial in developing interventions, messages, and reducing fear and stigma [38]. Furthermore, research indicated that training and employing local volunteer staff could increase public confidence and reduce the fear of disease stigma during the COVID-19 crisis [32, 39]. These results suggest that crisis management systems during epidemics require a people-centered approach to improve participation and promote healthy behaviors. The establishment of committees and local health teams with the participation of trusted volunteers and local leaders can raise community awareness and improve health education, thereby increasing public confidence in health decisions and reducing the fear of social stigma.

### Unintentional non-compliance

Insufficient or contradictory information, negligence, and feelings of inevitability are some of the factors that have a significant impact on people’s lack of participation in preventive measures against COVID-19. These factors have been categorized as unintentional non-compliance in the present study. Also, some of them unintentionally did not follow such behaviors for 3 reasons, including **insufficient or contradictory information, negligence** and **inevitability**.

#### Insufficient or contradictory information

Insufficient dissemination of information, particularly among the elderly and those with poor literacy skills, has resulted in non-compliance with health protocols. This problem has been compounded by constantly changing health guidelines and conflicting information on disease prevention, causing confusion among both employees and the general public. Consequently, trust in healthcare workers and related education has declined. This contention is supported by existing evidence. For instance, research conducted by Rasouli in Iran highlighted the difficulty of controlling COVID-19 owing to the absence of unified and comprehensive guidelines for mitigating the virus [26]. Additionally, a 2021 study conducted in Ireland revealed that low literacy levels among travelers were the primary impediment to sensitivity and response to COVID-19 [40].

A systematic review showed that different countries have adopted diverse measures to enhance access to adequate information regarding COVID-19 care and prevention. Thailand, for instance, has leveraged over one million local volunteers to disseminate and reinforce health information and education throughout various communities. Singapore has deployed volunteers to educate the elderly, while Liberia has succeeded in containing COVID-19 by empowering local leaders. Russia, also, has employed virtual methods and videos featuring celebrities to educate the masses about COVID-19 and its prevention [31]. Therefore, educational approaches tailored to the cultural and social circumstances of distinct communities should be utilized during epidemics to improve people’s access to adequate information. Thus, we recommend empowering neighborhood health liaisons in rural areas and trusted individuals in villages, including village councils and committees, to help convey sufficient information to the people during crises.

#### Inevitability

According to the participants, individuals were sometimes forced to disregard health protocols due to various reasons, including limited access to goods and health services, inadequate quarantine support, and the pursuit of administrative matters in person. These factors made it challenging to contain the disease, as adherence to social distancing and exposure reduction protocols, which are essential to minimize disease transmission, became impractical. Khankeh et al. (2021) reported similar findings in their study, highlighting how the absence of financial aid and national support programs in Iran impeded compliance with quarantine measures, resulting in continued social and business engagements [41]. A study conducted in South Africa similarly revealed that quarantine policies may drive individuals to hide from healthcare services and avoid diagnosis, particularly when their livelihoods are at stake [36].

In response to these challenges, the southern Indian state of Kerala utilized intersectoral approaches featuring volunteer participation to ensure the quarantine of sick individuals while meeting patients’ medical and non-medical needs [30]. The findings of a study carried out in Bangladesh in 2020 indicate that effective measures for quarantining individuals during an epidemic encompass resource distribution and provision of food assistance to the affected community and vulnerable populations [27]. Singapore also employed volunteers to distribute essential resources during the COVID-19 pandemic [31].

Presence in villages and health houses during the critical conditions of the pandemic accompanied by adherence to hygiene principles and COVID prevention measures made interviewing challenging for the interviewees. The high workload of healthcare workers and health educators as interviewers during the COVID-19 crisis caused service delivery to be the top priority over conducting interviews, thus taking considerable time for these interviews to take place. Recalling some difficult memories of the crisis period and the shortages upset the healthcare workers, leading them to sideline the interviews. Additionally, the long distances of some villages and the need to create safe and comfortable conditions for the healthcare workers to be able to express themselves calmly posed constraints on the implementation of the plan, considering the presence of patients in the health houses.

## Limitations

It was difficult for the interviewers to be present in the villages and health centers in the critical conditions of the pandemic along with the observance of hygiene principles and prevention of Covid. The busyness of Behvarzes and health trainers as interviewers during the Covid-19 crisis made providing services the first priority to conduct interviews, so it took a long time to interview these many health workers. Remembering some difficult memories during the crisis and shortages made Behvarzes uncomfortable, so they withdrew from the interview. Also, the study results may be subject to recall bias. The long distance of some villages and the creation of safe and comfortable conditions for Behvarzes to be able to express his thoughts in complete peace due to the presence of clients in the Health House were some of the limitations of the plan. The results may not be generalizable to all Iranian population.

## Conclusion

The present study showed valuable lessons from the recent pandemic. Based on the results, even with the efforts of healthcare workers, if other parts of the society and especially if the people themselves do not follow the health protocols, they will not be successful in epidemic control programs. People sometimes do not follow the health protocols because they consider themselves obliged to adhere to some cultural norms. Therefore, these habits are the main obstacle and educational programs at the community level should be developed to promote cultural habits related to prevention. Sometimes, due to reasons such as ignorance, they unintentionally neglect preventive behaviors. Therefore, the research findings suggest that crisis management requires attention to all factors contributing to its control, as well as intersectoral cooperation. A unidimensional approach and a complete transfer of disease management to the health sector tend to overlook other significant dimensions affecting the epidemic of diseases, such as cultural, and social aspects. In Iran, extensive sanctions and inadequate resources have led to insufficient aid for patients and vulnerable groups to access health and livelihood services during quarantine. Therefore, to enhance crisis management in Iran, it is essential to effectively utilize the capacities of civil society organizations and philanthropists, while ensuring proper organization of these capacities within the country.

## Data Availability

The datasets used and/or analyzed during the current study are available from the corresponding author upon reasonable request.
